# Structure-activity-relationship study of *N*-acyl-*N*-phenylpiperazines as potential inhibitors of the Excitatory Amino Acid Transporters (EAATs): improving the potency of a micromolar screening Hit is not truism

**DOI:** 10.1186/2193-1801-2-112

**Published:** 2013-03-14

**Authors:** Tri HV Huynh, Charles S Demmer, Bjarke Abrahamsen, Emil Marcher, Mikael Frykman, Anders A Jensen, Lennart Bunch

**Affiliations:** Department of Drug Design and Pharmacology, Faculty of Health and Medical Sciences, University of Copenhagen, Universitetsparken 2, Copenhagen, OE 2100 Denmark

**Keywords:** Excitatory amino acid transporters, EAATs, Rational ligand design, Medicinal chemistry

## Abstract

The excitatory amino acid transporters (EAATs) are transmembrane proteins responsible for the uptake of (*S*)-glutamate from the synaptic cleft. To date, five subtypes EAAT1-5 have been identified for which selective inhibitors have been discovered for EAAT1 and EAAT2. By screening of a commercially available compound library consisting of 4,000 compounds, *N*-acyl-*N*-phenylpiperazine analog **(±)-*exo*-1** was identified to be a non-selective inhibitor at EAAT1-3 displaying IC_50_ values in the mid-micromolar range (10 *μ*M, 40 *μ*M and 30 *μ*M at EAAT1, 2 and 3, respectively). Subsequently, we designed and synthesized a series of analogs to explore the structure-activity-relationship of this scaffold in the search for analogs characterized by increased inhibitory potency and/or EAAT subtype selectivity. Despite extensive efforts, all analogs of **(±)-*exo*-1** proved to be either inactive or to have least 3-fold lower inhibitory potency than the lead, and furthermore none of the active analogs displayed selectivity for a particular subtype amongst the EAAT1-3. On the basis of our findings, we speculate that **(±)-*exo*-1** binds to a recess (deepening) on the EAAT proteins than a well-defined pocket.

## Background

**Figure 1:**
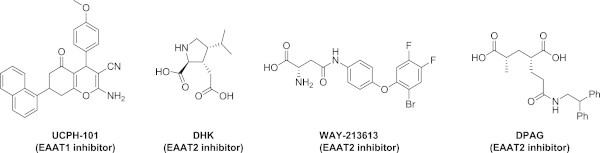
**Chemical structures of the EAAT1-selective inhibitor UCPH-101, (Jensen et al.** 2009 **; Erichsen et al.** 2010 **) and EAAT2-selective inhibitors DHK, (Jensen & Bräuner-Osborne** 2004 **) DPAG, (Sagot et al.** 2008 **) and WAY-213613 (Dunlop et al.** 2005 **).**

In the central nervous system (CNS), the excitatory amino acid transporters (EAATs) are transmembrane proteins responsible for the uptake of (*S*)-glutamate (Glu) from the synaptic cleft. Five subtypes have been identified, named EAAT1–EAAT5 in humans and GLAST, GLT-1, EAAC1, EAAT4 and EAAT5, respectively, in rodents. (Bunch et al. 2009) While EAAT5 is found exclusively in the retina, subtypes EAAT1–4 are expressed differentially within the CNS with respect to brain regions as well as at the cellular level: EAAT1 and EAAT2 are expressed primarily on astrocytes, but EAAT2 is also found in neurons, astrocytes and oligodendrocytes. (Lauriat et al. 2007) Subtype EAAT3 is distributed predominantly in postsynaptic neuronal sites, (Nieoullon et al. 2006) whereas EAAT4 is distributed in Purkinje cells as well as in the cerebral cortex. (Massie et al. 2001) Discovery of subtype selective ligands for the EAATs has attracted much attention over the past decade, (Jensen et al. 2009) the latest being the disclosure of UCPH-101 as first subtype selective EAAT1-inhibitor (Figure 1). (Jensen et al. 2009; Erichsen et al. 2010; Huynh et al. 2012,ab).

## Results and discussion

**Figure 2:**
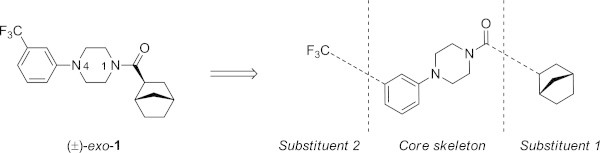
**Chemical structure of screening hit** ***N*** **-acyl-** ***N*** **-phenylpiperazine (±)-exo-1 displaying inhibitory activity at EAAT1-3 in the mid-micromolar range (IC**_**50**_ **= 10, 40 and 30** ***μ*** **M, respectively).**

From screening of a 4,000 compounds-library at HEK293 cells stably expressing human EAAT1-3 *N*-acyl-*N*-phenylpiperazine analog (±)**-***exo***-1** was identified as a non-selective inhibitor at the transporter exhibiting IC_50_ values in the mid-micromolar range (10 *μ*M, 40 *μ*M and 30 *μ*M at EAAT1, -2 and -3, respectively, Figure 2).

Although phenylpiperazines are promiscuous hits in high-throughput screenings (HTS) and a frequent core skeleton in marketed drugs, (Millan et al. 2001; Fragasso et al. 2006; Weisberg et al. 2007) we were motivated to explore the structure-activity-relationship (SAR) of this new class of EAAT inhibitors. A conventional medicinal chemistry analysis of (±)-*exo*-**1** suggests that the amide functionality, the aniline nitrogen, the phenyl ring and the trifluoromethyl group may play key roles in binding of this class of EAAT inhibitors. Consequently, the chemical structure can be broken down into three fragments: a core skeleton being the acyl-phenylpiperazine scaffold and two substituents being the trifluoromethyl- and the bicyclo[2.2.1]heptanyl group (Figure 1). The SAR study was designed as to study the influence of one of the two substituents (Figure 1) individually including the stereochemical organization around the α-carbonyl carbon.

**Scheme 1:**
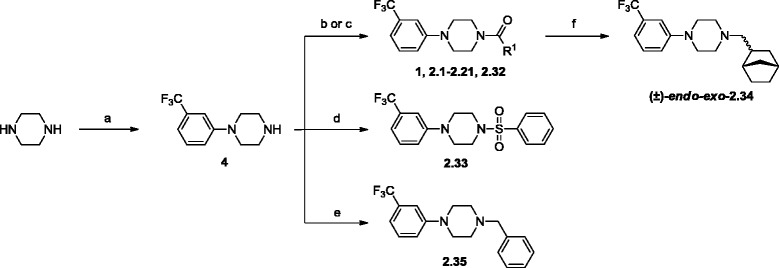
Synthesis of phenylpiperazine **4** and analogs **1, 2.1-2.21** and **2.33-2.35** ^a^. **Synthesis of phenylpiperazine 4 and analogs 1, 2.1-2.21 and 2.33-2.35** ^**a**^ **.** ^a^Reagents and conditions: (**a**) 3-Trifluoromethylphenylbromide, Pd(OAc)_2,_ P(^*t*^Bu)_3,_ NaO^*t*^Bu, *o*-xylene, 120°C, 17 h, 77%. (**b**) Appropriate carboxylic acid, TBTU, DIPEA, dry DMF, rt, 20 h, 51-92%. (**c**) Appropriate acid chloride (except for the synthesis of **2.32**: benzyl carbonochloridate), Et_3_N, dry dichloromethane, rt, 30 min, 71-78%. (**d**) Benzenesulfonyl chloride, Et_3_N, dry dichloromethane, rt, 30 min, 71%. (**e**) BnBr, Et_3_N, dry dichloromethane, rt, 24 h, 57%. (**f**) Starting from (±)-*endo-exo*-**1**: LiAlH_4_, THF, rt, 72 h, 71%.

**Scheme 2:**
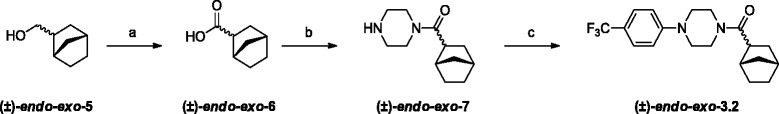
**Synthesis of piperazine analog (±)-** ***endo-exo*** **-3.2** ^**a**^ **.** ^a^ Reagents and conditions: (**a**) KMnO_4_, K_2_CO_3_, H_2_O, rt, 17 h. (**b**) Piperazine, DIPEA, TBTU, DMF, rt, 24 h, 49%. (**c**) 1-Bromo-4-(trifluoromethyl)benzene, Pd(OAc)_2_, P(^*t*^Bu)_3_, NaO^*t*^Bu, dry *o*-xylene, 120°C, 24 h, 56%.

The SAR study commenced by investigation of the influence of bicycle[2.2.1]heptanyl group on the EAAT inhibitory activity. The stereochemical configuration of the α-cabonyl carbon was addressed by the synthesis of *endo*-conformer (±)-*endo*-**1** (Table 1) from commercially available (±)-*endo*-carboxylic acid and *N*-(3-trifluoromethylphenyl)-piperazine **4**, the latter prepared by a palladium-catalyzed amination of commercially available piperazine (Scheme 1). (Nishiyama et al. 1998) Furthermore, the racemic and diastereomeric mixture (±)-*endo-exo*-**1** was prepared from the corresponding acid (±)-*endo-exo*-**6** obtained from oxidation of commercially available (±)-*endo-exo*-bicyclo[2.2.1]heptanylmethyl alcohol ((±)-*endo-exo*-**5**) (Scheme 2) using KMnO_4_ and K_2_CO_3_ in H_2_O. Isolation of (±)-*endo-exo*-**6** turned out to be difficult for which reason it was used directly in the next step. (Gudipati et al. 1993) To search for the optimal bulkiness of the lipophilic substituent, larger as well as smaller rigid hydrophobic ring-systems were introduced (**2.1-2.5**). Moreover, analogs **2.6**-**2.10** comprising alkyl group of varying length and bulkiness were designed to explore the effect of increased flexibility of this substituent on ligand binding. Furthermore, analogs **2.11–2.17** address if the bicyclo[2.2.1]heptane could be substituted for an aromatic moiety, whereas the analogs **2.18**–**2.21** were designed to explore the distinct substitution for a hydrophobic group. The synthesis of piperazine analogs **1** and **2.1**-**2.21** was carried out by amidation of **4** using the respective carboxylic acids, acid chlorides, benzenesulfonyl chloride and benzyl carbonochloridate afforded the corresponding amides in moderate to good yields (Scheme 1). The rationally designed 3-trifluoromethylphenylpiperazine analogs were supplemented by commercially available analogs **2.22**–**2.31**, as a quick way of expanding the SAR into the chemical space beyond rational guidance. Finally, the importance of the amide functionality was explored by the synthesis of carbamate **2.32** by acylation of **4** with carbonochloridate, sulfonamide **2.33** by treatment of **4** with phenylsulfonyl chloride, amine **2.34** by reduction of (±)-*endo-exo*-**1** with LiAlH_4_, (Cook et al. 1992) and *N*-benzyl analog **2.35**. by alkylation of phenylpiperazine **4** (Burkhard et al. 2010) In addition, these analogs were supplemented by five commercially available structurally diverse analogs **2.36**–**2.40**.

**Table 1:** **Pharmacological characterization of piperazine analogs 1 and 2.1-2.40 as inhibitors at HEK293 cells stably expressing human EAAT1-3 in the [** ^**3**^ **H]-D-aspartate uptake assay (Jensen & Bräuner-Osborne** 2004 **)**


	R ^1^	EAAT1	EAAT2	EAAT3
**(±)-** ***exo*** **-1**		10 [5.03 ± 0.12]	40 [4.44 ± 0.14]	30 [4.78 ± 0.10]
**(±)-** ***endo*** **-1**		14 [4.88 ± 0.09]	32 [4.52 ± 0.12]	10 [5.04 ± 0.13]
**(±)-** ***endo-exo*** **-1**		14 [4.87 ± 0.08]	42 [4.42 ± 0.11]	14 [4.94 ± 0.16]
**2.1**		>300	>300	>300
**2.2**		>300	>300	>300
**2.3**		~100	~100	~100
**2.4**		>300	>300	>300
**2.5**		>300	>300	>300
**2.6**		>1000	>300	>300
**2.7**		>1000	>100	>100
**2.8**		>300	>300	>300
**2.9**		>300	>300	>300
**2.10**		>300	>300	>300
**2.11**		~150	>100	>100
**2.12**		>100	>100	>100
**2.13**		>300	>300	>300
**2.14**		>300	>300	>300
**2.15**		>300	>300	>300
**2.16**		>100	>100	>100
**2.17**		>300	>300	>1000
**2.18**		>300	>300	>300
**2.19**		>300	>300	>300
**2.20**		>300	>300	>300
**2.21**		>300	>300	>300
**2.22**		~100	>100	>100
**2.23**		>300	>300	>100
**2.24**		>100	>100	>100
**2.25**		>300	>100	>300
**2.26**		>300	>300	>300
**2.27**		>300	>300	>300
**2.28**		>300	>300	>300
**2.29**		>300	>100	>100
**2.30**		>300	>1000	>300
**2.31**		>100	>100	>100
**2.32**		>1000	>300	>300
**2.33**		>1000	>300	>1000
**(±)-** ***endo-exo*** **-2.34**		>300	>300	>300
**2.35**		>300	>300	>1000
**2.36**		>300	>100	>300
**2.37**		>300	>100	>100
**2.38**		>300	>300	>300
**2.39**		>100	>100	>100
**2.40**		>500	>1000	>500

All values are given as IC_50_ in *μ*M with pIC_50_ ± S.E.M. values in brackets (for the active analogs).

**Scheme 3:**
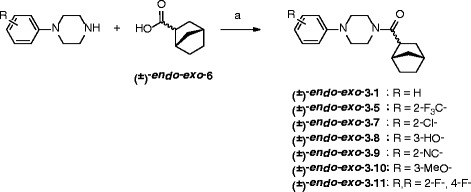
**Synthesis of piperazine analogs 3.1, 3.5, 3.8-3.11 by use of the coupling reagent TBTU** ^**a**^ **.** ^a^ Reagents and conditions: (**a**) TBTU, DIPEA, dry DMF, rt, 20 h, 40-58%.

We then turned to the design of analogs for investigation of the influence on EAAT inhibitory activity of the chemical nature of the trifluoromethyl group as well as its position on the phenyl ring. A series of 12 analogs were included in the SAR study, all wherein the (±)-*endo-exo*-bicyclic[2.2.1]-acyl group was conserved (analogs **3.1-3.12**, Table 2): Simplification of the chemical structure by depletion of the trifluoromethyl group provides analog (±)-*endo-exo*-**3.1**, while shifting the 3-trifluoromethyl group to the 4- or 2-positions affords analogs (±)-*endo-exo*-**3.2** and (±)-*endo-exo*-**3.5**, respectively (Table 2 and Scheme 2). The latter two analogs were supplemented by commercially available analogs (±)-*endo-exo-***3.3**, (±)-*endo-exo*-**3.4** and (±)-*endo-exo*-**3.6**. Continuing the design stage, substitution of the 3-trifluoromethyl group for a chloride, hydroxyl-, cyano- and methoxy group, provides analogs (±)-*endo-exo*-**3.7-3.10** respectively (Table 2), whereas 2,4-difluorophenyl analog (±)-*endo-exo*-**3.11** was included due to readily available starting materials. Analog (±)-*endo-exo*-**3.12** could be obtained from commercial suppliers and thus included with the notion that it comprises an *N*-diphenylmethyl group, which is indeed chemically distinct from the *N*-3-trifluoromethylphenyl group and furthermore the connecting nitrogen will be protonated at physiological pH=7.4. The analogs were synthesized starting from the appropriate phenylpiperazine and (±)-*endo-exo*-**6** under standard coupling conditions (TBTU, DIPEA in DMF) for the amide formation to afford the target compounds in moderate yields (Scheme 3) (Balalaie et al. 2007).

**Table 2:** **Pharmacological characterization of analogs 3.1–3.12 as inhibitors at HEK293 cells stably expressing human EAAT1-3 in the [** ^**1**^ **H]-D-aspartate uptake assay (Jensen & Bräuner-Osborne** 2004 **)**


	R ^2^	EAAT1	EAAT2	EAAT3
**(±)-** ***endo-exo*** **-3.1**		>300	>300	>300
**(±)-** ***endo-exo*** **-3.2**		>300	>300	>300
**(±)-** ***endo-exo*** **-3.3**		>100	>100	>100
**(±)-** ***endo-exo*** **-3.4**		>100	~100	>300
**(±)-** ***endo-exo*** **-3.5**		~300	>300	>300
**(±)-** ***endo-exo*** **-3.6**		>100	~100	>100
**(±)-** ***endo-exo*** **-3.7**		~300	~300	>300
**(±)-** ***endo-exo*** **-3.8**		>300	>300	>300
**(±)-** ***endo-exo*** **-3.9**		>300	>300	>300
**(±)-** ***endo-exo*** **-3.10**		>300	>300	>300
**(±)-** ***endo-exo*** **-3.11**		>300	>300	>300
**(±)-** ***endo-exo*** **-3n12**		>100	~100	>100

All values are given as IC_50_ in *μ*M with pIC_50_ ± S.E.M. values in brackets (for the active analogs).

### Pharmacological characterization

In total, 54 piperazine analogs **2.1**-**2.40** and **3.1**-**3.12** were characterized pharmacologically at stable HEK293 cells expressing human EAAT1-3 in a [^3^H]-D-aspartate uptake assay, (Jensen & Bräuner-Osborne 2004) and the results are summarized in Table 1 and Table 2. The *endo*-isomer **1** (*endo*-isomer) displayed inhibitory activities at EAAT1-3 comparable with those of the *exo*-isomer **1** (lead structure) (IC_50_ = 14 *μ*M, 32 *μ*M and 10 *μ*M vs. 10 *μ*M, 40 *μ*M and 30 *μ*M, respectively). In line with this, *endo*-*exo***1**, which is a 1:1 ratio of *endo*/*exo* moiety displayed IC_50_ values at EAAT1-3 of IC_50_ = 14 *μ*M, 42 *μ*M and 14 *μ*M, respectively. Usually, such findings would lead to the conclusion that the bicyclo-[2.2.1]-heptanyl group occupies a promiscuous hydrophobic pocket, which could be optimized for increased potency. However, upon increasing or decreasing the hydrophobic bulk and/or flexibility, a clear drop in potency was observed (analogs **2.1**-**2.17**, Table 1). Except for analogs **2.3** and **2.11**, which displayed only a 5-15 fold drop in inhibitory potency across the subtypes, all of these analogs would be characterized as *inactive* (IC_50_ >100 *μ*M or >300 *μ*M, Table 1). These findings could open up for the hypothesis that the pocket is indeed not hydrophobic but instead hydrophilic in nature. Upon binding of the hydrophobic alkane group, water molecules are forced out and ligand binding is entropically driven rather than enthalpically. Thus analogs **2.18**-**2.31**, which comprise a hydrophobic group, could be potential inhibitors. However, none of these displayed any inhibitory activity at EAAT1-3 (IC_50_ >100, >300 or >1000 *μ*M, Table 1). Continuing the characterization, neither the carbamate **2.32** nor the sulfonamide **2.33** analog displayed inhibitory activity at EAAT1-3, and likewise all amines **2.34-2.40** were found to be inactive at all three subtypes.

The pharmacological results for the twelve analogs **3.1-3.12,** which address the influence of substituent 2 (Figure 2) on inhibitory activity at EAAT1-3 are summarized in Table 2. While it was not surprising that removal or repositioning of the 3-trifluoromethyl group (analog **3.1**, **3.2** and **3.5**, respectively) resulted in loss of inhibitory activity, the further nine analogs **3.3**, **3.4**, **3.6**-**3.12** were also inactive or displayed at least a 3-fold lower inhibitory activity than (±)**-*exo*-1**.

## Conclusion

In conclusion, screening of a compound library identified (±)-*exo***-1** as a broad range EAAT1-3 inhibitor exhibiting IC_50_ values at the three transporters in the mid-micromolar range. Subsequently, rational design and synthesis of 33 analogs of (±)-*exo***-1** was carried out, together with the purchase of 21 analogs. Thus, a total of 54 piperazine analogs were characterized pharmacologically as inhibitors at EAAT1-3 but only the *endo* diastereomer (±)-*endo***-1** displayed inhibitory potency in the mid-micromolar range comparable to that of the lead structure (±)-*exo***-1**. The remaining analogs were inactive or at least three fold weaker inhibitors at EAAT1-3 than the lead, none of them displaying signs of subtype-selectivity. Given the structural diversity of the analogs characterized pharmacologically, we speculate if the lead structure (±)-*exo***-1** adheres to a recess (deepening) in the surface of the protein rather than binds in an organized way to a well-defined pocket.

### Experimental section

All commercially available reagents were used without further purification. THF was distilled over sodium/benzophenone, Et_2_O was dried over neatly cut sodium and dichloromethane was dried over 3 Å molecular sieves. All solvents were tested for water content using a Karl Fisher apparatus. All reactions involving dry solvents or sensitive agents were performed under a nitrogen atmosphere, and glassware was dried prior to use. All reactions were monitored by analytical thin-layer chromatography (TLC, Merck silica gel 60 F_254_ aluminum sheets). Flash chromatography was carried out using Merck silica gel 60A (35-70 micron). ^1^H NMR spectra were recorded on a 300 MHz Varian Mercury 300BB or a 400 MHz Avance Bruker and ^13^C NMR spectra on a 75 MHz Varian Gemini 2000BB or a 100 MHz Avance Bruker. Preparative HPLC was done using Agilent Prep HPLC systems with Agilent 1100 series pump, Agilent 1200 series diode array, multiple wavelength detector (G1365B), and Agilent PrepHT High Performance Preparative Cartridge Column (Zorbax, 300 SB-C18 Prep HT, 21.2 × 250 mm, 7 *μ*m). LC-MS spectra were recorded using an Agilent 1200 series solvent delivery system equipped with an autoinjector coupled to an Agilent 6400 series triple quadrupole mass spectrometer equipped with an electrospray ionization source. Gradients of 5% aqueous MeCN + 0.05% HCOOH (eluent A), and 95% aqueous MeCN + 0.043% formic acid (eluent B) were employed. Melting points were measured using a MPA 100 Optimelt automatic melting point system and are stated uncorrected. Compounds were dry either under high vacuum or freeze dried using a Holm & Halby, Heto LyoPro 6000 freezedrier.

#### General procedure a: synthesis of amides using O-benzotriazole-1-yl-N,N,N',N'-tetramethyluronium tetrafluoroborate (TBTU) as coupling reagent

To a suspension of the appropriate phenylpiperazine analog (0.33 mmol), the carboxylic acid (0.40 mmol) and TBTU (0.43 mmol) in dry DMF (4 mL) under an N_2_ atmosphere, was added DIPEA (1.32 mmol) and reaction mixture was stirred for 20 h at rt. The reaction mixture was quenched with brine (5 mL) and extracted with dichloromethane (3 × 20 mL). The combined organic phases were washed with H_2_O (20 mL) and brine (20 mL) and dried over anhydrous Na_2_SO_4_. After concentration *in vacuo*, the crude product was purified by column chromatography on silica gel in accordance with details described for the analog.

#### General procedure B: synthesis of amides using acid chlorides

To a suspension of 1-(3-(trifluoromethyl)phenyl)piperazine (**4**) (0.33 mmol) in dry dichloromethane (5 mL) at 0°C under a N_2_ atmosphere was added Et_3_N (0.91 mmol). The reaction mixture was stirred for 10 min at 0°C, then the appropriate acid chloride (0.48 mmol) was added and stirring continued for 30 minutes at rt. The reaction mixture was quenched with sat. NH_4_Cl (5 mL) and extracted with dichloromethane (3 × 20 mL). The combined organic phases were washed with H_2_O (20 mL) and brine (20 mL) and dried over Na_2_SO_4_. After concentration *in vacuo*, the crude product was purified by column chromatography on silica gel in accordance with details described for the analog.

### (±)-*endo-*Bicyclo[2.2.1]heptan-2-yl(4-(3-(trifluoromethyl)phenyl)piperazin-1-yl)methanone ((±)-*endo-*1)

Obtained from 1-(3-(trifluoromethyl)phenyl)piperazine (**4**) and commercially available (±)-*endo*-bicyclo[2.2.1]heptane-2-carboxylic acid by general procedure **A** in 61% yield as a pale-yellow oil. *R*_f_ 0.25 (heptane/EtOAc 3:1). ^1^H NMR (300 MHz, CDCl_3_) *δ* 7.36 (t, *J* = 8.4 Hz, 1H), 7.12-7.04 (m, 3H), 3.94-3.89 (m, 1H), 3.79-3.62 (m, 3H), 3.30-3.08 (m, 4H), 2.95 (dt, *J* = 10.8, 4.2 Hz, 1H), 2.40 (br s, 1H), 2.29 (br s, 1H), 1.95 (ddd, *J* = 12.0, 4.5, 2.4 Hz, 1H), 1.64-1.28 (m, 7H). ^13^C NMR (75 MHz, CDCl_3_) *δ* 172.3, 151.1, 131.5 (q, *J* = 31.5 Hz), 129.7, 124.2 (q, *J* = 270.7 Hz), 119.3, 116.5 (q, *J* = 3.8 Hz), 112.6 (q, *J* = 3.8 Hz), 49.5, 45.2, 43.7, 41.7, 40.8, 40.5, 40.2, 37.1, 32.2, 29.1, 21.1. LC-MS (*m/z*) calcd for C_19_H_23_F_3_N_2_O [M+H^+^], 353.2; found, 353.2. Anal. calcd for C_19_H_23_F_3_N_2_O × 1HCl: C 58.69, H 6.22, N 7.20 found C 59.28, H 6.24, N 7.18.

### (±)-*endo- exo*-Bicyclo[2.2.1]heptan-2-yl(4-(3-(trifluoromethyl)phenyl)piperazin-1-yl)methanone ((±)-*endo-exo-*1)

Prepared from 1-(3-(trifluoromethyl)phenyl)piperazine (**4**) and (±)-*endo-exo-*bicyclo[2.2.1]heptane-2-carboxylic acid ((±)-*endo-exo-*(**6**)) by general procedure **A** in 36% yield as a pale-yellow oil. *R*_f_ 0.50 (heptane/EtOAc 1:1). ^1^H NMR (300 MHz, CDCl_3_) *δ* 7.38-7.33 (m, 1H), 7.12-7.02 (m, 3H), 3.98-3.96 (m, 1H), 3.75 (br s, 2H), 3.67 (br s, 2H), 3.21 (br s, 4H), 2.98-2.92 (m, 0.6H), 2.40 (br s, 1.4H), 2.29 (br s, 1H), 1.95-1.92 (m, 1H), 1.62-1.25 (m, 6H). ^13^C NMR (75 MHz, CDCl_3_) *δ* 173.1, 151.3, 131.5 (q, *J* = 31.5 Hz), 129.7, 124.2 (q, *J* = 270.7 Hz), 119.2, 116.5 (q, *J* = 3.8 Hz), 112.7 (q, *J* = 3.8 Hz), 50.2, 49.8, 45.2, 43.7, 41.7, 40.8, 40.4, 36.7, 32.3, 28.9, 21.9. LC-MS (*m/z*) calcd for C_19_H_23_F_3_N_2_O [M+H^+^], 353.2; found, 353.4. HPLC: purity_254_ > 99%.

### Bicyclo[2.2.2]octan-2-yl(4-(3-(trifluoromethyl)phenyl)piperazin-1-yl)methanone (2.1)

Obtained from 1-(3-(trifluoromethyl)phenyl)piperazine (**4**) and racemic (±)-*endo-exo*-bicyclo[2.2.2]octane-2-carboxylic acid ((±)-*endo-exo*-(**6**)) by general procedure **A** in 59% yield as a pale-yellow oil. *R*_f_ 0.95 (1:1 heptane/EtOAc). ^1^H NMR (400 MHz, CDCl_3_) *δ* 7.12 (t, *J* = 6.0 Hz, 1H), 6.50 (dd, *J* = 6.0, 1.0 Hz, 1H), 6.42-6.35 (m, 2H), 3.82 (br s, 2H), 3.64 (br s, 2H), 3.32 (br s, 4H), 2.79-2.76 (m, 1H), 2.19-2.16 (m, 1H), 1.79-1.73 (m, 1H), 1.69-1.18 (m, 10H). ^13^C NMR (100 MHz, CDCl_3_) *δ* 174.0, 151.2, 131.5 (q, *J* = 31.5 Hz), 129.7, 125.6 (q, *J* = 270.7 Hz), 119.3, 116.5 (q, *J* = 3.8 Hz), 112.6 (q, *J* = 3.8 Hz), 49.2, 45.2, 41.6, 38.8, 28.4, 27.6, 26.5, 25.3, 25.2, 23.9, 21.5. LC-MS (*m/z*) calcd for C_20_H_25_F_3_N_2_O [M+H^+^], 366.2; found, 366.2. HPLC: purity_254_ > 98%.

### Adamantan-1-yl(4-(3-(trifluoromethyl)phenyl)piperazin-1-yl)methanone (2.2)

Prepared from 1-(3-(trifluoromethyl)phenyl)piperazine (**4**) and 1-adamantanecarboxylic acid by general procedure **A** in 73% yield as a pale-yellow oil. *R*_f_ 0.68 (100% EtOAc). ^1^H NMR (300 MHz, CDCl_3_) *δ* 7.39-7.32 (m, 1H), 7.12-7.04 (m, 3H), 3.87 (dd, *J* = 6.0, 3.0 Hz, 4H), 3.21 (dd, *J* = 6.0, 3.0 Hz, 4H), 2.06-2.03 (m, 9H), 1.70-1.77 (m, 6H). ^13^C NMR (75 MHz, CDCl_3_) *δ* 176.2, 151.5, 131.1 (q, *J* = 31.5 Hz), 130.1, 127.2 (q, *J* = 270.8 Hz), 119.4, 116.9 (q, *J* = 3.8 Hz), 112.8 (q, *J* = 3.8 Hz), 55.2, 49.7, 49.6, 45.4, 43.9, 42.1, 39.5, 37.1, 28.9, 19.1. LC-MS (*m/z*) calcd for C_22_H_27_F_3_N_2_O [M+H^+^], 393.2; found, 393.2. Anal. calcd for C_22_H_27_F_3_N_2_O × 1HCl: C 61.61, H 6.58, N 6.53 found C 61.38, H 6.38, N 6.38.

### Cyclohexyl(4-(3-(trifluoromethyl)phenyl)piperazin-1-yl)methanone (2.3)

Prepared from 1-(3-(trifluoromethyl)phenyl)piperazine (**4**) and cyclohexanecarboxylic acid by general procedure **A** in 59% yield as a yellow oil. *R*_f_ 0.15 (heptane/EtOAc 3:1). ^1^H NMR (300 MHz, CDCl_3_) *δ* 7.35 (t, *J* = 7.8 Hz, 1H), 7.12-7.03 (m, 3H), 3.78 (br s, 2H), 3.67 (br s, 2H), 3.21 (br s, 4H), 2.50 (tt, *J* = 11.4, 3.3 Hz, 1H), 1.83-1.69 (m, 5H), 1.55 (dq, *J* = 11.4, 3.9 Hz, 2H), 1.35-1.23 (m, 3H). ^13^C NMR (75 MHz, CDCl_3_) *δ* 175.0, 151.5, 131.9 (q, *J* = 31.5 Hz), 130.1, 124.6 (q, *J* = 270.8 Hz) 119.7 (q, *J* = 1.5 Hz), 117.0 (q, *J* = 3.8 Hz), 113.0 (q, *J* = 3.8 Hz), 49.8, 49.5, 45.1, 40.9, 29.8, 26.3. LC-MS (*m/z*) calcd for C_18_H_23_F_3_N_2_O [M+H^+^], 341.1; found, 341.1. Anal. calcd for C_18_H_23_F_3_N_3_O × 1HCl: C 57.37, H 6.42, N 7.43 found C 57.38, H 6.20, N 7.38.

### Cyclopentyl(4-(3-(trifluoromethyl)phenyl)piperazin-1-yl)methanone (2.4)

Prepared from 1-(3-(trifluoromethyl)phenyl)piperazine (**4**) and cyclopentanecarbonyl chloride by general procedure **B** in 85% yield as a yellow oil. *R*_f_ 0.50 (heptane/EtOAc 1:1). ^1^H NMR (400 MHz, CDCl_3_) *δ* 7.37 (t, *J* = 8.0 Hz, 1H), 7.13-7.06 (m, 3H), 3.80 (dd, *J* = 8.0, 4.0 Hz, 2H), 3.70 (dd, *J* = 8.0, 4.0 Hz, 2H), 3.24 (dd, *J* = 8.0, 4.0 Hz, 2H ), 3.19 (dd, *J* = 8.0, 4.0 Hz, 2H), 1.92-1.54 (m, 9H). ^13^C NMR (100 MHz, CDCl_3_) *δ* 176.1, 147.0, 134.2 (q, *J* = 31.5 Hz), 132.4, 125.0 (q, *J* = 270.7 Hz), 124.9, 118.1 (q, *J* = 3.8 Hz), 117.6 (q, *J* = 3.8 Hz), 54.7, 43.1, 42.4, 31.6, 27.5. LC-MS (*m/z*) calcd for C_17_H_21_F_3_N_2_O [M+H^+^], 327.2; found, 327.2. HPLC: purity_254_ > 97%.

### Cyclopropyl(4-(3-(trifluoromethyl)phenyl)piperazin-1-yl)methanone (2.5)

Prepared from 1-(3-(trifluoromethyl)phenyl)piperazine (**4**) and cyclopropanecarboxylic acid by general procedure **A** in 51% yield as a yellow oil. *R*_f_ 0.55 (100% EtOAc). ^1^H NMR (300 MHz, CDCl_3_) *δ* 7.39-7.32 (m, 1H), 7.12-7.05 (m, 3H), 3.83 (br s, 4H), 3.26 (br s, 2H), 3.24 (br s, 2H), 1.78 (m, 1H), 1.05-1.00 (m, 2H), 0.84-0.78 (m, 2H). ^13^C NMR (75 MHz, CDCl_3_) *δ* 172.5, 151.5, 131.9 (q, *J* = 31.5 Hz), 130.1, 127.5 (q, *J* = 270.8 Hz), 119.6 (q, *J* = 0.8 Hz), 116.9 (q, *J* = 3.8 Hz), 113.0 (q, *J* = 3.8 Hz), 49.5, 49.3, 45.6, 42.2, 11.4, 8.0. LC-MS (*m/z*) calcd for C_15_H_17_F_3_N_2_O [M+H^+^], 299.1; found, 299.1. Anal. calcd for C_15_H_17_F_3_N_2_O × 1HCl: C 53.82, H 5.42, N 8.37 found C 56.15, H 5.15, N 8.17.

### 2,2-Dimethyl-1-(4-(3-(trifluoromethyl)phenyl)piperazin-1-yl)propan-1-one (2.6)

Prepared from 1-(3-(trifluoromethyl)phenyl)piperazine (**4**) and pivaloyl chloride by general procedure **B** in 78% yield as a clear oil. *R*_f_ 0.28 (heptane/EtOAc 3:1). ^1^H NMR (300 MHz, CDCl_3_) *δ* 7.35 (dt, *J* = 8.1, 0.9 Hz, 1H), 7.11-7.03 (m, 3H), 3.81 (dd, *J* = 5.1, 5.1 Hz, 4H), 3.21 (dd, *J* = 5.1, 5.1 Hz, 4H), 1.32 (s, 9H). ^13^C NMR (75 MHz, CDCl_3_) *δ* 176.4, 151.1, 131.5 (q, *J* = 31.5 Hz), 129.7, 124.2 (q, *J* = 270.8 Hz), 119.1 (q, *J* = 1.5 Hz), 116.5 (q, *J* = 3.8 Hz), 112.4 (q, *J* = 3.8 Hz), 49.0, 44.8, 38.7, 28.4. LC-MS (*m/z*) calcd for C_16_H_21_F_3_N_2_O [M+H^+^], 315.2; found, 315.2. Anal. calcd for C_16_H_21_F_3_N_2_O × 1HCl: C 55.11, H 6.21, N 7.96 found C 54.78, H 6.32, N 7.99.

### 1-(4-(3-(Trifluoromethyl)phenyl)piperazin-1-yl)ethanone (2.7)

Obtained from 1-(3-(trifluoromethyl)phenyl)piperazine (**4**) and acetic acid by general procedure **A** in 64% yield as a yellow oil. *R*_f_ 0.22 (100% EtOAc). ^1^H NMR (300 MHz, CDCl_3_) *δ* 7.36 (t, *J* = 9.0 Hz, 1H), 7.13-7.04 (m, 3H), 3.78 (dd, *J* = 6.0, 3.0 Hz, 2H), 3.64 (dd, *J* = 6.0, 3.0 Hz, 2H), 3.24 (dd, *J* = 6.0, 3.0 Hz, 2H), 3.20(dd, *J* = 6.0, 3.0 Hz, 2H), 2.15 (s, 3H). ^13^C NMR (75 MHz, CDCl_3_) *δ* 169.4, 151.4, 131.9 (q, *J* = 31.5 Hz), 130.1, 124.6 (q, *J* = 270.8 Hz), 119.7 (q, *J* = 1.5 Hz), 117.1 (q, *J* = 3.8 Hz), 113.1 (q, *J* = 3.8 Hz), 49.5, 49.3, 46.4, 41.6, 21.8. LC-MS (*m/z*) calcd for C_13_H_15_F_3_N_2_O [M+H^+^], 273.1; found, 273.1. Anal. calcd for C_13_H_15_F_3_N_2_O × 1HCl: C 50.58, H 5.22, N 9.07 found C 50.73, H 5.02, N 8.74.

### 3-Methyl-1-(4-(3-(trifluoromethyl)phenyl)piperazin-1-yl)butan-1-one (2.8)

Prepared from 1-(3-(trifluoromethyl)phenyl)piperazine (**4**) and isovaleryl chloride by general procedure **B** in 59% yield as a pale-yellow oil. *R*_f_ 0.40 (heptane/EtOAc 1:1). ^1^H NMR (400 MHz, CDCl_3_) *δ* 7.39 (t, *J* = 6.9 Hz, 1H), 7.16-7.10 (m, 3H), 3.82 (dd, *J* = 6.0, 3.0 Hz, 2H), 3.68 (dd, *J* = 6.0, 4.0 Hz, 2H), 3.23 (br s, 4H), 2.27 (d, *J* = 4.0 Hz, 2H), 2.17 (septet, *J* = 6.0 Hz, 1H), 1.00 (d, *J* = 6.0 Hz, 6H). ^13^C NMR (100 MHz, CDCl_3_) *δ* 173.7, 147.8, 133.4 (q, *J* = 31.5 Hz), 132.1, 123.9, 123.6 (q, *J* = 270.8 Hz), 117.1 (q, *J* = 3.8 Hz), 113.4 (q, *J* = 3.8 Hz), 47.8, 42.6, 41.3, 27.0, 22.9. LC-MS (*m/z*) calcd for C_16_H_21_F_3_N_2_O [M+H^+^], 315.2; found, 315.2. HPLC: purity_254_ > 99%.

### 3,3-Dimethyl-1-(4-(3-(trifluoromethyl)phenyl)piperazin-1-yl)butan-1-one (2.9)

Prepared from 1-(3-(trifluoromethyl)phenyl)piperazine (**4**) and 3,3-dimethylbutanoyl chloride by general procedure B in 73% yield as a pale-orange oil. *R*_f_ 0.50 (heptane/EtOAc 1:1). ^1^H NMR (400 MHz, CDCl_3_) *δ* 7.39-7.35 (m, 1H), 7.14-7.11 (m, 2H), 7.08-7.06 (m, 1H), 3.81 (dd, *J* = 8.0, 4.0 Hz, 2H), 3.69 (dd, *J* = 8.0, 4.0 Hz, 2H), 3.22 (dd, *J* = 8.0, 4.0 Hz, 2H), 3.21 (dd, *J* = 8.0, 4.0 Hz, 2H), 2.31 (s, 2H), 1.08 (s, 9H). ^13^C NMR (100 MHz, CDCl_3_) *δ* 171.9, 147.3, 134.2 (q, *J* = 31.5 Hz), 132.3, 126.2, 124.7 (q, *J* = 270.8 Hz), 123.4 (q, *J* = 3.8 Hz), 117.4 (q, *J* = 3.8 Hz), 54.5, 46.1, 33.0, 31.5, 21.1. LC-MS (*m/z*) calcd for C_17_H_23_F_3_N_2_O [M+H^+^], 329.2; found, 329.2. HPLC: purity_254_ > 96%.

### 4-Methyl-1-(4-(3-(trifluoromethyl)phenyl)piperazin-1-yl)pentan-1-one (2.10)

Prepared from 1-(3-(trifluoromethyl)phenyl)piperazine (**4**) and 4-methylvaleryl chloride by general procedure **B** in 87% yield as a clear oil. *R*_f_ 0.35 (heptane/EtOAc 1:1). ^1^H NMR (400 MHz, CDCl_3_) *δ* 7.37 (t, *J* = 6.0 Hz, 1H), 7.14-7.07 (m, 3H), 3.79 (dd, *J* = 8.0, 4.0 Hz, 2H), 3.65 (dd, *J* = 8.0, 4.0 Hz, 2H), 3.24 (dd, *J* = 8.0, 4.0 Hz, 2H), 3.22 (dd, *J* = 8.0, 4.0 Hz, 2H), 2.35 (dd, *J* = 6.0, 6.0 Hz, 2H), 1.63-1.52 (m, 3H), 0.93 (d, *J* = 3.0 Hz, 6H). ^13^C NMR (100 MHz, CDCl_3_) *δ* 172.1, 151.1, 131.6 (q, *J* = 31.5 Hz), 129.7, 124.2 (q, *J* = 271.0 Hz), 119.3, 116.6 (q, *J* = 4.0 Hz), 112.7 (q, *J* = 4.0 Hz), 49.2, 49.0, 45.4, 41.3, 34.2, 31.3, 27.9, 22.7, 22.4. LC-MS (*m/z*) calcd for C_17_H_23_F_3_N_2_O [M+H^+^], 329.2; found, 329.2. HPLC: purity_254_ > 99%.

### Phenyl(4-(3-(trifluoromethyl)phenyl)piperazin-1-yl)methanone (2.11)

Prepared from 1-(3-(trifluoromethyl)phenyl)piperazine (**4**) and benzoic acid by general procedure **A** in 58% yield as a pale-yellow oil. *R*_f_ 0.33 (heptane/EtOAc 2:3). ^1^H NMR (300 MHz, CDCl_3_) *δ* 7.47-7.39 (m, 6H), 7.26-7.20 (m, 2H), 7.10 (d, *J* = 7.5 Hz, 1H), 3.75 (br s, 2H), 3.48 (br s, 2H), 3.28 (br s, 4H). ^13^C NMR (75 MHz, CDCl_3_) *δ* 170.8, 151.5, 135.8, 132.1 (q, *J* = 31.5 Hz), 130.3 (d, *J* = 1.5 Hz), 130.1 (d, *J* = 1.5 Hz), 129.0, 127.5, 124.6 (q, *J* = 267.0 Hz), 119.9, 117.2, 113.3, 49.7, 47.8. LC-MS (*m/z*) calcd for C_18_H_17_F_3_N_2_O [M+H^+^], 335.1; found, 335.1. Anal. calcd for C_18_H_17_F_3_N_2_O × 1HCl: C 58.30, H 4.89, N 7.55 found C 58.20, H 4.78, N 7.43.

### Benzo[*d*][1,3]dioxol-5-yl(4-(3-(trifluoromethyl)phenyl)piperazin-1-yl)methanone (2.12)

Obtained from 1-(3-(trifluoromethyl)phenyl)piperazine (**4**) and benzo[*d*][1,3]dioxole-5-carboxylic acid by general procedure **A** in 60% yield as a white solid. *R*_f_ 0.18 (heptane/EtOAc 3:1). ^1^H NMR (300 MHz, CDCl_3_) *δ* 7.36 (t, *J* = 9.0 Hz, 1H), 7.13-7.05 (m, 3H), 6.97-6.82 (m, 3H), 6.00 (s, 2H), 3.77 (br s, 4H), 3.24 (br s, 4H). ^13^C NMR (75 MHz, CDCl_3_) *δ* 169.9, 151.0, 149.0, 147.7, 131.5 (q, *J* = 31.5 Hz), 129.7, 128.9, 124.1 (q, *J* = 270.8 Hz), 121.7, 119.4, 116.8 (q, *J* = 3.8 Hz), 112.8 (q, *J* = 3.8 Hz), 108.2, 108.1, 101.5, 49.2, 42.8; mp 87-89°C. LC-MS (*m/z*) calcd for C_19_H_17_F_3_N_2_O_3_ [M+H^+^], 379.1; found, 379.1. Anal. calcd for C_19_H_17_F_3_N_2_O_3_: C 60.32, H 4.53, N 7.40 found C 59.95, H 4.40, N 7.02.

### (3-Phenoxyphenyl)(4-(3-(trifluoromethyl)phenyl)piperazin-1-yl)methanone (2.13)

Prepared from 1-(3-(trifluoromethyl)phenyl)piperazine (**4**) and 3-phenoxybenzoic acid by general procedure **A** in 62 % yield as a pale-yellow oil. *R*_f_ 0.20 (heptane/EtOAc 3:1). ^1^H NMR (300 MHz, CDCl_3_) *δ* 7.40-7.22 (m, 5H), 7.16-6.99 (m, 8H), 3.90 (br s, 2H), 3.60 (br s, 2H), 3.25 (br s, 2H), 3.16 (br s, 2H). ^13^C NMR (75 MHz, CDCl_3_) *δ* 170.1, 158.1, 156.7, 151.4, 137.4, 132.0 (q, *J* = 31.5 Hz), 130.6, 130.4, 130.1, 124.6 (q, *J* = 270.8 Hz), 124.4, 121.9, 120.2, 119.9, 119.6, 117.3 (q, *J* = 3.8 Hz), 113.3 (q, *J* = 3.8 Hz), 49.7, 42.9. LC-MS (*m/z*) calcd for C_24_H_21_F_3_N_2_O_2_ [M+H^+^], 427.1; found, 427.1. Anal. calcd for C_24_H_21_F_3_N_3_O_2_ × 1HCl: C 62.27, H 4.79, N 6.05 found C 63.52, H 4.64, N 5.90.

### 1-Phenylcyclopentyl-(4-(3-(trifluoromethyl)phenyl)piperazin-1-yl)methanone (2.14)

Prepared from 1-(3-(trifluoromethyl)phenyl)piperazine (**4**) and 1-phenylcyclopentanecarboxylic acid by general procedure **A** in 92 % yield as an off-white solid. *R*_f_ 0.37 (heptane/EtOAc 3:1). ^1^H NMR (300 MHz, CDCl_3_) *δ* 7.40-7.17 (m, 8H), 7.05 (d, *J* = 6.0 Hz, 1H), 3,78 (br s, 4H), 3.25 (br s, 2H), 3.13 (br s, 2H), 2.70-2.41 (m, 4H), 2.06-1.87 (m, 4H). ^13^C NMR (75 MHz, CDCl_3_) *δ* 175.2, 151.4, 145.8, 143.2, 131.9 (q, *J* = 31.5 Hz), 130.0, 128.7, 127.5, 126.8, 124.6 (q, *J* = 270.8 Hz) 119.4 (q, *J* = 1.5 Hz), 116.8 (q, *J* = 3.8 Hz), 112.9 (q, *J* = 3.8 Hz), 59.3, 58.9, 48.7, 38.8, 36.4, 25.7, 24.0; mp 77-79°C. LC-MS (*m/z*) calcd for C_23_H_25_F_3_N_2_O [M+H^+^], 403.2; found, 403.2. Anal. calcd for C_23_H_25_F_3_N_2_O: C 68.64, H 6.26, N 6.96 found C 70.90, H 6.37, N 6.73.

### Naphthalen-1-yl(4-(3-(trifluoromethyl)phenyl)piperazin-1-yl)methanone (2.15)

Prepared from 1-(3-(trifluoromethyl)phenyl)piperazine (**4**) and 1-naphthoic acid by general procedure **A** in 83% yield as a colorless oil. *R*_f_ 0.19 (heptane/EtOAc 3:1). ^1^H NMR (300 MHz, CDCl_3_) *δ* 7.90-7.83 (m, 3H), 7.55-7.32 (m, 5H), 7.12-7.02 (m, 3H), 4.21-4.01 (m, 2H), 3.42-3.35 (m, 4H), 3.11-2.99 (m, 2H). ^13^C NMR (75 MHz, CDCl_3_) *δ* 169.9, 151.4, 134.1, 133.9, 132.0 (q, *J* = 31.5 Hz), 130.1, 129.8, 128.9, 127.6, 127.0, 125.6, 125.1, 124.5 (q, *J* = 270.8 Hz), 124.3, 119.4 (q, *J* = 1.5 Hz), 117.3 (q, *J* = 3.8 Hz), 113.3 (q, *J* = 3.8 Hz), 50.1, 49.7, 47.3, 42.0. LC-MS (*m/z*) calcd for C_22_H_19_F_3_N_2_O [M+H^+^], 385.1; found, 385.1. Anal. calcd for C_22_H_19_F_3_N_2_O × 1HCl: C 62.79, H 4.79, N 6.66 found C 64.82, H 4.72, N 6.26.

### Thiophen-2-yl(4-(3-(trifluoromethyl)phenyl)piperazin-1-yl)methanone (2.16)

Prepared from 1-(3-(trifluoromethyl)phenyl)piperazine (**4**) and thiophene-2-carboxylic acid by general procedure **A** in 69% yield as a pale-yellow oil. *R*_f_ 0.68 (100% EtOAc). ^1^H NMR (300 MHz, CDCl_3_) *δ* 7.46 (dd, *J* = 6.0, 3.0 Hz, 1H), 7.39-7.32 (m, 2H), 7.13-7.04 (m, 4H), 3.92 (dd, *J* = 6.0, 3.0 Hz, 4H), 3.28 (dd, *J* = 6.0, 3.0 Hz, 4H). ^13^C NMR (75 MHz, CDCl_3_) *δ* 164.1, 151.4, 137.1, 131.9 (q, *J* = 31.5 Hz), 130.1, 129.5, 129.3, 127.2, 124.6 (q, *J* = 270.8 Hz), 119.7 (q, *J* = 1.5 Hz), 117.2 (q, *J* = 3.8 Hz), 113.1 (q, *J* = 3.8 Hz), 49.5, 45.7. LC-MS (*m/z*) calcd for C_16_H_15_F_3_N_2_OS [M+H^+^], 341.0; found, 341.0. Anal. calcd for C_16_H_15_F_3_N_2_OS ×1HCl: C 51.00, H 4.28, N 7.43 found C 51.41, H 4.28, N 7.33.

### 1-(4-(3-(Trifluoromethyl)phenyl)piperazin-1-yl)-2-phenylethanone (2.17)

Prepared from 1-(3-(trifluoromethyl)phenyl)piperazine (**4**) and 2-phenylacetyl chloride by general procedure **B** in 77% yield as a clear oil. *R*_f_ 0.28 (heptane/EtOAc 1:1). ^1^H NMR (300 MHz, CDCl_3_) *δ* 7.34-7.20 (m, 6H), 7.09-6.96 (m, 3H), 3.79 (dd, *J* = 6.0, 3.0 Hz, 2H), 3.78 (s, 2H), 3.58 (dd, *J* = 6.0, 3.0 Hz, 2H), 3.16 (dd, *J* = 6.0, 3.0 Hz, 2H), 2.99 (dd, *J* = 6.0, 3.0 Hz, 2H). ^13^C NMR (75 MHz, CDCl_3_) *δ* 169.8, 151.3, 137.8, 131.3 (q, *J* = 31.5 Hz), 129.4, 129.1, 128.3, 127.1, 124.3 (q, *J* = 270.8 Hz), 118.6 (d, *J* = 0.8 Hz), 115.6 (q, *J* = 3.8 Hz), 112.0 (q, *J* = 3.8 Hz), 62.9, 52.8, 48.6. LC-MS (*m/z*) calcd for C_19_H_19_F_3_N_2_O [M+H^+^], 349.1; found, 349.1. Anal. Calcd for C_19_H_19_F_3_N_2_O × 1HCl: C 60.20, H 5.16, N 7.12 found C 59.30, H 5.24, N 7.28.

### 4-Aminophenyl-(4-(3-(trifluoromethyl)phenyl)piperazin-1-yl)methanone (2.18)

Prepared from 1-(3-(trifluoromethyl)phenyl)piperazine (**4**) (103 mg, 0.37 mmol) and 4-((*tert*-butoxycarbonyl)amino)benzoic acid in accordance with general procedure A to provide *N*-Boc-**2.18** in 48% yield as a brown oil. *R*_f_ 0.44 (heptane/EtOAc 1:1). ^1^H NMR (400 MHz, MeOD) *δ* 7.37-7.27 (m, 5H), 7.07-6.99 (m, 3H), 3.81-3.61 (m, 4H), 3.27-3.07 (m, 4H), 1.46 (s, 9H). ^13^C NMR (100 MHz, MeOD) *δ* 170.3, 152.5, 151.1, 140.1, 131.8, 131.5, 129.7, 128.5, 127.5, 125.6, 119.4, 118.1, 116.8, 114.1, 112.9 (d, *J* = 4.0 Hz), 81.1, 49.2, 31.9, 29.0, 28.3, 22.7. LC-MS (*m/z)* calcd for C_23_H_26_F_3_N_3_O_3_ [M+H^+^], 450.1, found, 450.1. The intermediate product *N*-Boc-**2.18** was dissolved in DCM (2 mL) and TFA (2 mL) and stirred for 1h at rt. The reaction mixture was evaporated and the title compound **2.18** was obtained in 69% yield as TFA-salt. ^1^H NMR (400 MHz, MeOD) *δ* 7.43-7.39 (m, 1H), 7.35-7.32 (m, 2H), 7.23-7.20 (m, 2H), 7.12-7.10 (m, 1H), 6.90-6.87 (m, 2H), 3.79 (s, 4H), 3.30-3.26 (m, 4H). ^13^C NMR (100 MHz, MeOD) *δ* 173.1, 152.1, 148.3, 132.8, 132.5 (q, *J* = 8.0 Hz), 131.0, 130.4, 127.2, 126.7, 124.5, 120.8, 118.6, 117.2 (q, *J* = 1.0 Hz), 117.0, 115.0, 113.6 (q, *J* = 1.0 Hz), 50.1, 49.7. LC-MS (*m/z)* calcd for C_18_H_18_F_3_N_3_O [M+H^+^], 350.1; found, 350.1. HPLC: purity_254_ >97%.

### 3-Aminophenyl-(4-(3-(trifluoromethyl)phenyl)piperazin-1-yl)methanone (2.19)

Prepared from 3-trifluoromethylphenylpiperazine (**4**) (101 mg, 0.37 mmol) and 3-((*tert*-butoxycarbonyl)amino)benzoic acid by general procedure **A** in 85% yield as a reddish oil. *R*_f_ 0.42 (heptane/EtOAc 1:1). ^1^H NMR (400 MHz, MeOD) *δ* 7.45 (s, 1H), 7.36-7.23 (p, *J* = 8.0 Hz, 3H), 7.06-6.98 (m, 4 H), 6.78 (s, 1H), 3.95-3.75 (m, 2H), 3.66-3.46 (m, 2H), 3.27-3.01 (m, 4H), 1.45 (s, 9H). ^13^C NMR (100 MHz, MeOD) *δ* 172.4, 155.2, 152.9, 141.1 (q, *J* = 2.0 Hz), 137.2, 132.6 (q, *J* = 4.0 Hz), 131.0, 130.3, 129.8, 127.2, 124.8, 122.0, 120.9 (q, *J* = 2.0 Hz), 118.0, 117.3, 113.7, 81.2, 79.5, 54.8, 30.7, 28.7, 24.3, 23.8. LC-MS (*m/z)* calcd for C_23_H_26_F_3_N_3_O_3_ [M+H^+^], 450.1, found, 450.1. The intermediate product *N*-Boc-**2.19** was dissolved in DCM (2 mL) and TFA (2 mL) and stirred for 1h at rt. The reaction mixture was evaporated to afford the title compound in 99% yield as TFA-salt. ^1^H NMR (400 MHz, MeOD) *δ* 7.67-7.62 (m, 2H), 7.57-7.49 (m, 3H), 7.44-7.40 (m, 1H), 7.24-7.22 (m, 2H), 7.14-7.12 (m, 1H), 4.01-3.80 (m, 2H), 3.74-3.50 (m, 2H), 3.45-3.18 (m, 4H). ^13^C NMR (100 MHz, MeOD) *δ* 170.8, 159.0 (q, *J* = 10.5 Hz), 152.7, 138.6, 134.6, 134.24, 133.5, 132.5 (q, *J* = 8.0 Hz), 131.7, 131.5, 131.01, 130.6, 128.5, 127.5, 127.2, 125.0, 124.9, 124.5, 122.3, 120.9 (d, *J* = 1.0 Hz), 117.4 (q, *J* = 1.0 Hz), 114.7, 113.7 (q, *J* = 1.0 Hz), 54.5, 54.2. LC-MS (*m/z)* calcd for C_18_H_18_F_3_N_3_O [M+H^+^], 350.1; found, 350.1. HPLC: purity_254_ >96%.

### 2-Amino-1-(4-(3-(trifluoromethyl)phenyl)piperazin-1-yl)ethanone (2.20)

Prepared from **(4)** (104 mg, 0.37 mmol) and *N*-Boc-glycine-OH (105 mg, 0.36 mmol) by general procedure A to provide *N*-Boc-**2.20** in 86% yield as a colorless oil. *R*_f_ 0.25 (heptane/EtOAc 1:1). ^1^H NMR (400 MHz, MeOD) *δ* 7.41 (t, *J* = 8.0 Hz, 1H), 7.23-7.20 (m, 2H), 7.11 (d, *J* = 8.0 Hz, 1H), 3.97 (s, 2H), 3.75 (s, 2H), 3.66 (s, 2H), 3.28-3.23 (m, 4H), 1.45 (s, 9H). The intermediate *N*-Boc-**2.20** was dissolved in DCM (2 mL) and TFA (2 mL) and stirred for 30 min at rt. The reaction mixture was evaporated to afford the title compound as the TFA-salt in quantitative yield. ^1^H NMR (400 MHz, MeOD) *δ* 7.45-7.40 (m, 1H), 7.24-7.21 (m, 2H), 7.14-7.12 (m, 1H), 4.00 (s, 2H), 3.80 (t, *J* = 4.0 Hz, 2H), 3.62 (t, *J* = 4.0 Hz, 2H), 3.30-3.26 (m, 2H). ^13^C NMR (100 MHz, MeOD) *δ* 165.7, 159.9 (q, *J* = 41.0 Hz), 152.7, 132.40 (t, 32.0 Hz), 131.0, 127.2, 124.5, 120.9, 117.5, 114.7, 113.6 (q, *J* = 4.0 Hz), 49.9, 49.7, 45.5, 43.1, 41.0, 27.7. LC-MS (*m/z)* calcd for C_13_H_16_F_3_N_3_O [M+H^+^], 288.1; found, 288.1. HPLC: purity_254_ >99%.

### 2-Amino-1-(4-(3-(trifluoromethyl)phenyl)piperazin-1-yl)ethanone (2.21)

Prepared from **(16)** (103 mg, 0.37 mmol) and *N*-Boc-glycine-OH (69 mg, 0.36 mmol) by general procedure A to provide *N*-Boc-**2.21** in 51% yield as a colorless oil. *R*_f_ 0.18 (heptane/EtOAc 1:1). The intermediate *N*-Boc-**2.20** was dissolved in DCM (2 mL) and TFA (2 mL) and stirred for 45 min at rt. The reaction mixture was evaporated to afford the title compound as the TFA-salt in quantitative yield. ^1^H NMR (400 MHz, MeOD) *δ* 7.44-7.40 (m, 1H), 7.24-7.20 (m, 2H), 7.12 (dt, *J* = 8.0 Hz, 1H), 3.78 (t, *J* = 4.0 Hz, 2H), 3.68 (t, *J* = 4.0, 2H), 3.26-3.21 (m, 6H), 2.83 (t, *J* = 4.0 Hz, 2H). ^13^C NMR (100 MHz, MeOD) *δ* 170.4, 152.8, 132.7, 132.4, 131.0, 127.2, 120.8, 117.5, 117.3 (q, *J* = 4.0 Hz), 113.5 (q, *J* = 4.0 Hz), 54.4, 50.0, 46.16, 42.65. LC-MS (*m/z)* calcd for C_14_H_18_F_3_N_3_O [M+H^+^], 302.1; found, 302.1. HPLC: purity_254_ >98%.

### Benzyl 4-(3-(trifluoromethyl)phenyl)piperazine-1-carboxylate (2.32)

Prepared from 1-(3-(trifluoromethyl)phenyl)piperazine (**4**) and benzyl carbonochloridate by general procedure **B** in 73% yield as a clear oil. *R*_f_ 0.43 (heptane/EtOAc 3:1). ^1^H NMR (300 MHz, CDCl_3_) *δ* 7.36-7.29 (m, 6H), 7.10-7.01 (m, 3H), 5.15 (s, 2H), 3.66 (dd, *J* = 6.0, 3.0 Hz, 4H), 3.17 (br s, 4H). ^13^C NMR (75 MHz, CDCl_3_) *δ* 155.1, 151.2, 136.5, 131.3 (q, *J* = 31.5 Hz), 129.6, 128.5, 128.1, 127.9, 124.2 (q, *J* = 270.8 Hz), 119.4 (q, *J* = 1.5 Hz), 116.5 (q, *J* = 3.8 Hz), 112.4 (q, *J* = 3.8 Hz), 67.3, 48.9, 43.5. LC-MS (*m/z*) calcd for C_19_H_19_F_3_N_2_O_2_ [M+H^+^], 365.1; found, 365.1. Anal. Calcd for C_19_H_19_F_3_N_2_O_2_ × 1HCl: C 57.35, H 5.03, N 6.91 found C 56.93, H 5.03, N 6.99.

### 1-(Phenylsulfonyl)-4-(3-(trifluoromethyl)phenyl)piperazine (2.33)

Prepared from 1-(3-(trifluoromethyl)phenyl)piperazine (**4**) and benzenesulfonyl chloride by general procedure **B** in 71% yield as a white solid. *R*_f_ 0.39 (heptane/EtOAc 3:1). ^1^H NMR (300 MHz, CDCl_3_) *δ* 7.80-7.76 (m, 2H), 7.65-7.51 (m, 3H), 7.32 (t, *J* = 7.5 Hz, 1H), 7.11-6.97 (m, 3H), 3.30-3.26 (m, 4H), 3.19-3.16 (m, 4H). ^13^C NMR (75 MHz, CDCl_3_) *δ* 151.1, 135.6, 133.5, 131.9 (q, *J* = 31.5 Hz), 130.2, 129.6, 128.2, 124.5 (q, *J* = 270.8 Hz), 119.9 (q, *J* = 1.5 Hz), 117.4 (q, *J* = 3.8 Hz), 113.5 (q, *J* = 3.8 Hz), 49.1, 46.4; mp 109-111°C (decomposed). LC-MS (*m/z*) calcd for C_17_H_17_F_3_N_2_O_2_S [M+H^+^], 371.1; found, 371.1. Anal. Calcd for C_17_H_17_F_3_N_2_O_2_S: C 55.13, H 4.63, N 7.56 found C 54.60, H 4.44, N 7.41.

### (±)-*endo-exo*-Bicyclo[2.2.1]heptan-2-ylmethyl)-4-(3-(trifluoromethyl)phenyl)piperazine (2.34)

A solution of (±)-*endo-exo*-bicyclo[2.2.1]heptan-2-yl(4-(3-(trifluoromethyl)phenyl)piperazin-1-yl)methanone ((±)-*endo-exo***2.3**) (116.5 mg, 0.30 mmol) in dry THF (3 mL) was added dropwise to a solution of LiAlH_4_ (13 mg, 0.33 mmol) in THF (5 mL) at 0°C under a N_2_ atmosphere. The reaction mixture was stirred for 3 days at rt, quenched with 2N NaOH (2 mL) and extracted with dichloromethane (3 × 25 mL). The combined organic phases were washed with H_2_O (20 mL) and brine (20 mL). The organic phase was dried over anhydrous Na_2_SO_4_. After concentration *in vacuo,* the crude product was purified by column chromatography on silica gel to afford the titled compound as a pale-yellow oil (72 mg, 0.21 mmol, 71% yield): *R*_f_ 0.42 (heptane/EtOAc 2:1). ^1^H NMR (400 MHz, CDCl_3_) *δ* 7.37 (t, *J* = 8.0 Hz, 1H), 7.13-7.00 (m, 3H), 3.91-3.66 (m, 4H), 3.25-3.16 (m, 4H), 2.97-2.41 (m, 2H), 2.32-2.28 (m, 1H), 1.98-1.92 (m, 1H), 1.62-1.19 (m, 9H). ^13^C NMR (100 MHz, CDCl_3_) *δ* 151.2, 130.1 (q, *J* = 31.5 Hz), 130.2, 124.5 (q, *J* = 270.7 Hz), 119.3 (d, *J* = 3.8 Hz), 117.3 (q, *J* = 3.8 Hz), 113.2 (q, *J* = 3.8 Hz), 49.0, 44.3, 40.9, 40.4, 37.2, 36.7, 36.0, 34.9, 32.2, 29.5, 28.9, 24.5. LC-MS (*m/z)* calcd for C_19_H_25_F_3_N_2_ [M+H^+^], 339.2; found, 339.2. HPLC: purity_254_ > 99%.

### 1-Benzyl-4-(3-(trifluoromethyl)phenyl)piperazine (2.35)

BnBr (54 *μ*L, 0.46 mmol) was added dropwise to a solution of 1-(3-(trifluoromethyl)phenyl)piperazine (**4**) (100 mg, 0.43 mmol) and Et_3_N (126 *μ*L, 0.46 mmol) in dichloromethane (5 mL) at rt under a N_2_ atmosphere. The reaction mixture was stirred for 24 hours, quenched with saturated NH_4_Cl (5 mL) and extracted with dichloromethane (3 × 20 mL). The combined organic phases were washed with H_2_O (20 mL) and brine (20 mL). The organic phase was dried over anhydrous Na_2_SO_4_. After concentration *in vacuo,* the crude product was purified by column chromatography on silica gel to afford the titled compound as a clear oil (79 mg, 0.25 mmol, 57% yield): *R*_f_ 0.41 (heptane/EtOAc 2:1). ^1^H NMR (300 MHz, CDCl_3_) *δ* 7.34-7.19 (m, 6H), 7.08-6.99 (m, 3H), 3.55 (br s, 2H), 3.21 (dd, *J* = 6.0, 6.0 Hz, 4H), 2.59 (dd, *J* = 6.0, 6.0 Hz, 4H). ^13^C NMR (75 MHz, CDCl_3_) *δ* 151.3, 135.2, 131.7 (q, *J* = 31.5 Hz), 130.1, 129.3, 128.9, 127.4 124.6 (q, *J* = 270.8 Hz), 119.6 (d, *J* = 3.8 Hz), 117.0 (q, *J* = 3.8 Hz), 113.0 (q, *J* = 3.8 Hz), 49.3, 49.1, 46.2, 41.9, 41.5. LC-MS (*m/z*) calcd for C_18_H_19_F_3_N_2_ [M+H^+^], 321.2; found, 321.2. Anal. Calcd for C_18_H_19_F_3_N_2_ × 1HCl: C 60.59, H 5.65, N 7.85 found C 55.74, H 5.23, N 7.12.

### Bicyclo[2.2.1]heptan-2-yl(4-phenylpiperazin-1-yl)methanone (3.1)

Prepared in accordance with *general procedure A*. The crude product was purified by flash chromatography to afford **3.1** as a clear oil (240 mg, 43%). *R*_f_ 0.20 (heptane/EtOAc 3:1). ^1^H NMR (400 MHz, CDCl3 ) *δ* 7.31-7.25 (m, 2H), 6.95-6.85 (m, 3H), 3.99-3.61 (m, 4H), 3.30-3.03 (m, 4H), 2.98-2.91 (m, 0.5H), 2.44-2.39 (m, 1.5H), 2.34-2.26 (m, 1H), 1.99-1.90 (m, 1H), 1.64-1.16 (m, 7H). ^13^C NMR (100 MHz, CDCl3) *δ* 173.9, 172.2, 151.0, 129.2, 120.5, 120.5, 116.6, 50.2, 49.8, 49.7, 49.6, 45.4, 45.3, 44.2, 43.7, 41.9, 41.7, 40.8, 40.6, 40.4, 37.2, 36.7, 36.0, 34.9, 32.2, 29. 5, 28.9, 28.9, 24.5. LC-MS (*m/z)* calcd for C_18_H_24_N_2_O [M+H^+^], 285.1; found, 285.1. HPLC: purity_254_ >95%.

### (±)-*endo-exo*-Bicyclo[2.2.1]heptan-2-yl(4-(4-(trifluoromethyl)phenyl)piperazin-1-yl)methanone (3.2)

Pd(OAc)_2_ (1.0 mg, 4.6 *μ*mol) and P(^*t*^Bu)_3_ (1.0 M in toluene, 16 *μ*L, 0.016 mmol) were added to a solution of 4-(bicyclo[2.2.1]heptane-2-carbonyl)piperazin-1-ium chloride (±)-*endo-exo-***7** (101 mg, 0.41 mmol), 1-bromo-4-(trifluoromethyl)benzene (63 *μ*L, 0.45 mmol) and NaO^*t*^Bu (86 mg, 0.90 mmol) in dry *o*-xylene (1.3 mL) at rt under a N_2_ atmosphere. The reaction mixture was stirred at 120°C for 24 hours, quenched with H_2_O (10 mL) and extracted with EtOAc (3 × 20 mL). The combined organic phases were washed with H_2_O (20 mL) and brine (20 mL). The organic phase was dried over anhydrous Na_2_SO_4_. After concentration *in vacuo,* the crude product was purified by column chromatography on silica gel to afford the titled compound as a white solid (80 mg, 0.23 mmol, 56%): *R*_f_ 0.25 (heptane/EtOAc 3:1). ^1^H NMR (400 MHz, CDCl_3_) *δ* 7.42 (d, *J =* 8.6 Hz, 2H), 6.86 (d, *J =* 8.8 Hz, 2H), 3.90-3.80 (m, 0.5H), 3.75-3.62 (m, 4H), 3.31-3.08 (m, 4H), 2.91-2.80 (m, 0.5H), 2.40-2.30 (m, 1H), 2.28-2.19 (m, 1H), 1.91-1.82 (m, 1H), 1.60-1.10 (m, 7H). ^13^C NMR (100 MHz, CDCl_3_) *δ* 174.0, 172.3, 153.0, 126.6, 126.5, 126.4, 126.4, 125.9, 123.2, 121.4, 121.1, 114.9, 48.8, 48.4, 48.3, 48.2, 45.1, 44.9, 44.2, 43.7, 41.6, 41.4, 40.8, 40.3, 37.1, 36.7, 36.0, 34.9, 32.2, 29.5, 28.9, 28.9, 24.5; mp: 126-127°C (decomposed). LC-MS (*m/z*) calcd for C_19_H_23_F_3_N_2_O [M+H^+^], 353.2; found, 353.2. HPLC: purity_254_ > 95%.

### (±)-*endo-exo*-Bicyclo[2.2.1]heptan-2-yl(4-(2-(trifluoromethyl)phenyl)piperazin-1- yl)methanone ((±)-*endo-exo*-3.5)

Prepared in accordance with *general procedure A*. The crude product was purified twice by flash chromatography to afford (±)-*endo-exo***-3.5** as a clear oil (48 mg, 14%). *R*_f_ 0.32 (heptane/EtOAc 3:1). ^1^H NMR (400 MHz, CDCl3 ) *δ* 7.64 (d, *J =* 7.86 Hz, 1H), 7.52 (t, *J =* 7.69 Hz, 1H), 7.31 (d, *J =* 8.02 Hz, 1H), 7.28-7.22 (m, 1H), 3.72 (s, 4H), 2.99-2.85 (m, 4.5H), 2.45-2.36 (m, 1.5H) 2.33-2.25 (m, 1H), 2.00-1.90 (m, 1H), 1.65-1.15 (m, 7H). ^13^C NMR (100 MHz, CDCl3) *δ* 173.9, 172.3, 151.8, 132.8, 127.6, 127.3, 127.2, 125.2, 124.0, 122.6, 54.1, 53.8, 53.3, 53.2, 46.0, 45.9, 44.2, 43.7, 42.4, 42.2, 40.8, 40.6, 40.3, 37.2, 36.7, 36.0, 34.9, 32.2, 29.4, 28.9, 28.9, 24.5. LC-MS (*m/z*) calcd for C_19_H_23_F_3_N_2_O [M+H^+^], 353.1; found, 353.1. HPLC: purity_254_ > 95%.

### (±)-*endo-exo*-Bicyclo[2.2.1]heptan-2-yl(4-(3-chlorophenyl)piperazin-1-yl)methanone ((±)-*endo-exo*-3.7)

Prepared in accordance with *general procedure A*. The crude product was purified by flash chromatography to afford **3.7** as a clear oil (90 mg, 46%). *R*_f_ 0.31 (heptane/EtOAc 3:1). ^1^H NMR (400 MHz, CDCl3 ) *δ* 7.17 (t, *J =* 8.09 Hz, 1H), 6.87 (t, *J =* 2.04 Hz, 1H), 6.85 (d, *J =* 7.84 Hz, 1H) 6.78 (dd, *J =* 8.35, 2.15 Hz, 1H), 3.95-3.60 (m, 4H), 3.25-3.05(m, 4H), 2.97-2.90 (m, 0.5H), 2.45-2.37 (m, 1.5H), 2.33-2.25 (m, 1H) 1.98-1.88 (m, 1H), 1.64-1.06 (m, 7H). ^13^C NMR (100 MHz, CDCl3) *δ* 173.8, 172.1, 151.9, 134.9, 130.0, 119.9, 116.2, 114.3, 49.4, 49.1, 48.9, 45.1, 44.9, 44.9, 43.6, 41.5, 41.3, 40.7, 40.5, 40.2, 37.0, 36.6, 35.9, 34.8, 32.0, 29.3, 28.7, 24.4. LC-MS (*m/z*) calcd for C_18_H_23_ClN_2_O [M+H^+^], 319.1; found, 319.1. HPLC: purity_254_ > 97%.

### (±)-*endo-exo-*Bicyclo[2.2.1]heptan-2-yl(4-(3-hydroxyphenyl)piperazin-1-yl)methanone ((±)-*endo-exo*-3.8)

Prepared from (±)-*endo-exo*-bicyclo[2.2.1]heptane-2-carboxylic acid ((±)-*endo-exo***-6**) and 3-(piperazin-1-yl)phenol by general procedure **A** in 40% yield as a white solid. *R*_f_ 0.45 (heptane/EtOAc 2:1). ^1^H NMR (400 MHz, CDCl_3_) *δ* 7.13 (t, *J* = 8.0 Hz, 1H), 6.52-6.36 (m, 3H), 3.93-3.64 (m, 4H), 3.22-2.92 (m, 4H), 2.45-2.41 (m, 1H), 2.31-2.28 (m, 1H), 1.97-1.88 (m, 1H), 1.63-1.18 (m, 8H). ^13^C NMR (100 MHz, CDCl_3_) *δ* 173.5, 157.4, 152.3, 130.1, 108.7, 107.7, 103.6, 50.2, 49.8, 49.1, 45.3, 44.3, 43.7, 41.8, 40.6, 40.5, 37.2, 36.8, 36.0, 35.1, 32.2, 29.5, 28.9, 28.9, 24.5; mp: 187-189°C (decomposed). LC-MS (*m/z*) calcd for C_18_H_24_N_2_O_2_ [M+H^+^], 301.2; found, 301.2. HPLC: purity_254_ > 98%.

### 3-(4-(bicyclo[2.2.1]heptane-2-carbonyl)piperazin-1-yl)benzonitrile (3.9)

Prepared in accordance with general procedure A. The crude product was purified by flash chromatography to afford **3.9** as a clear oil (63 mg, 63%). *R*_f_ 0.15 (heptane/EtOAc 3:1). ^1^H NMR (400 MHz, CDCl3 ) *δ* 7.37-7.31(m, 1H), 7.15-7.10 (m, 3H), 3.98-3.62 (m, 4H), 3.30-3.10 (m, 4H), 2.98-2.90 (m, 0.5H), 2.46-2.36 (m, 1.5H), 2.34-2.26 (m, 1H), 1.98-1.88 (m, 1H), 1.64-1.10 (m, 7H). ^13^C NMR (100 MHz, CDCl3) *δ* 173.9, 172.3, 151.0, 130.0, 123.3, 120.4, 119.1, 118.9, 113.2, 49.1, 48.8, 48.6, 45.0, 44.9, 44.2, 43.7, 41.5, 41.3, 40.8, 40.6, 40.3, 37.1, 36.7, 36.0, 34.9, 32.2, 29.4, 28.9, 28.8, 24.5. LC-MS (*m/z*) calcd for C_19_H_23_N_3_O [M+H^+^], 310.1; found, 310.1. HPLC: purity_254_ > 99%.

### (±)-*endo-exo*-Bicyclo[2.2.1]heptan-2-yl(4-(3-methoxyphenyl)piperazin-1-yl)methanone ((±)-*endo-exo*-3.10)

Prepared from (±)-*endo-exo-*bicyclo[2.2.1]heptane-2-carboxylic acid ((±)-*endo-exo***-6**) and 1-(3-methoxyphenyl)piperazine by general procedure **A** in 45% yield as a clear oil. *R*_f_ 0.35 (heptane/EtOAc 2:1). ^1^H NMR (400 MHz, CDCl_3_) *δ* 7.19 (t, *J* = 8.0 Hz, 1H), 6.55-6.44 (m, 3H), 3.92-3.61 (m, 4H), 3.48 (s, 3H), 3.24-2.92 (m, 4H), 2.44-2.40 (m, 1H), 2.31-2.27 (m, 1H), 1.97-1.89 (m, 1H), 1.61-1.17 (m, 8H). ^13^C NMR (100 MHz, CDCl_3_) *δ* 173.2, 160.7, 152.4, 129.9, 109.3, 105.1, 103.1, 55.2, 50.8, 50.0, 49.6, 49.6, 49.4, 45.4, 45.3, 44.3, 43.8, 41.9, 41.7, 40.8, 40.6, 40.4, 37.2, 36.8, 36.0, 34.9, 32.2, 29.5, 29.0, 28.9, 27.0, 24.5. LC-MS (*m/z)* calcd for C_19_H_26_N_2_O_2_ [M+H^+^], 315.2; found, 315.2. HPLC: purity_254_ > 98%.

### (±)-*endo-exo*-Bicyclo[2.2.1]heptan-2-yl(4-(2,4-difluorophenyl)piperazin-1-yl)methanone ((±)-*endo-exo*-3.11)

Prepared from (±)-*endo-exo*-bicyclo[2.2.1]heptane-2-carboxylic acid ((±)-*endo-exo***-6**) and 1-(2,4-difluorophenyl)piperazine by general procedure A. After chromatography (*R*_f_ = 0.43, heptane/EtOAc 2:1). The title compound was isolated in 58% yield as a clear oil. ^1^H NMR (400 MHz, CDCl_3_) *δ* 6.92-6.77 (m, 3H), 3.93-3.64 (m, 4H), 3.07-2.90 (m, 4H), 2.44-2.27 (m, 2H), 1.98-1.90 (m, 1H), 1.62-1.17 (m, 8H). ^13^C NMR (100 MHz, CDCl_3_) *δ* 173.9, 172.3, 159.5 (d, *J* = 12.0 Hz), 157.0 (d, *J* = 12.0 Hz), 154.6 (d, *J* = 12.0 Hz), 136.4 (d, *J* = 4.0 Hz), 136.2 (d, *J* = 4.0 Hz), 129.0, 128.4, 126.9, 119.9 (q, *J* = 4.0 Hz), 110.9 (d, *J* = 2.0 Hz), 110.7 (d, *J* = 2.0 Hz), 105.1, 104.9 (d, 2.0 Hz), 104.6, 53.4, 51.8, 51.4, 51.0, 45.7, 45.6, 44.2, 43.2, 42.0, 41.8, 40.8, 40.6, 40.4, 37.2, 36.8, 36.0, 29.0, 28.9, 24.6. LC-MS (*m/z)* calcd for C_18_H_22_F_2_N_2_O [M+H^+^], 321.2; found, 321.2. HPLC: purity_254_ > 97%.

### 1-(3-(Trifluoromethyl)phenyl)piperazine (4)

Piperazine (5.74 g, 66.6 mmol), 3-bromobenzotrifluoride (5.0 g, 22.2 mmol), Pd(OAc)_2_ (50 mg, 220 *μ*mol), P(^*t*^Bu)_3_ (216 *μ*L, 880 *μ*mol) and NaO^*t*^Bu (3 g, 31.1 mmol) were stirred in dry *o*-xylene (50 mL) at 120ºC under a N_2_ atmosphere for 17 h. H_2_O (25 mL) was added and the crude reaction was extracted with EtOAc (3 × 50 mL) and the combined organic phases were washed with H_2_O (30 mL) and brine (30 mL). The organic phase was dried over anhydrous Na_2_SO_4_. After concentration *in vacuo*, the crude product was purified by column chromatography on silica gel. This afforded the titled compound (3.91 g, 17.0 mmol, 77%) as a yellow oil: *R*_f_ 0.23 (Et_2_O/MeCN/MeOH/Et_3_N 10:1:1:0.5). ^1^H NMR (300 MHz, CDCl_3_) *δ* 7.33 (t, *J* = 7.95 Hz, 1H), 7.10-7.03 (m, 3H), 3.18 (dd, *J* = 9.0, 5.4 Hz, 4H), 3.04 (dd, *J* = 6.9, 5.1 Hz, 4H), 2.0 (s, 1H). ^13^C NMR (75 MHz, CDCl_3_) *δ* 152.2, 131.9 (q, *J* = 30.8 Hz), 129.9, 124.7 (q, *J* = 271.5 Hz), 119.1 (q, *J* = 1.5 Hz), 116.2 (q, *J* = 3.8 Hz), 112.5 (q, *J* = 4.5 Hz), 50.1, 46.3. LC-MS (*m/z*) calcd for C_11_H_13_F_3_N_2_ [M+H^+^], 231.1; found, 231.1. HPLC: purity_254_ > 95%.

### (±)-*endo-exo*-Bicyclo[2.2.1]heptane-2-carboxylic acid ((±)-*endo-exo*-6)

A solution of KMnO_4_ (53.2 g, 336.5 mmol) in H_2_O (190 mL) was added dropwise to a solution of bicyclo[2.2.1]heptan-2-ylmethanol (17.0 g, 134.6 mmol) and K_2_CO_3_ (7.4 g, 53.8 mmol) in H_2_O (380 mL) at 0°C and stirred at room temperature for 24 hours. The crude reaction was quenched with 4N HCl (pH ≈ 2) and extracted with EtOAc (3 × 1000 mL). The combined organic phases were washed with H_2_O (500 mL), brine (250 mL) and dried over anhydrous MgSO_4_. After concentration *in vacuo,* the crude product was directly used for the next step without purification.

### (±)-*endo-exo*-Bicyclo[2.2.1]heptan-2-yl(piperazin-1-yl)methanone ((±)-*endo-exo-*7)

DIPEA (2.8 g, 21.4 mmol) was added dropwise to a stirred solution of (±)-*endo-exo*-bicyclo[2.2.1]heptane-2-carboxylic acid ((±)-*endo-exo***-7**) (1 g, 7.1 mmol) and piperazine (1.85 g, 21.4 mmol) in dry DMF (60 mL) at 0°C under a N_2_ atmosphere. A solution of TBTU (2.98 g, 9.27 mmol) in DMF (26 mL) was added to the reaction mixture and stirred at 0°C for 30 min. After 20 hours at rt, the reaction mixture was quenched with brine (30 mL) and extracted with dichloromethane (3 × 100mL). The combined organic phases were washed with H_2_O (3 × 200 mL) and brine (100 mL). The organic phase was dried over anhydrous Na_2_SO_4_. After concentration *in vacuo,* the crude product was purified by column chromatography on silica gel (Et_2_O/MeOH/MeCN/Et_3_N 8:2:2:0.5). This afforded the title compound as a pale-yellow oil (0.73 mg, 3.5 mmol, 49% yield). The pure product was dissolved in dichloromethane (40 mL) and 4N HCl in dioxane (0.88 mL, 3.5 mmol) was added. The solution was evaporated to afford the corresponding HCl salt. ^1^H NMR (300 MHz, CDCl_3_) *δ* 10.10 (br s, 2H), 4.10-3.80 (m, 4H), 3.22 (br s, 4H), 2.95-2.83 (m, 0.5H), 2.40-2.27 (m, 2.5H), 1.97-1.80 (m, 1H), 1.70-1.16 (m, 6H). ^13^C NMR (75 MHz, CDCl_3_) *δ* 173.8, 172.2, 45.9, 45.4, 45.2, 45.0, 44.9, 44.8, 44.0, 43.5, 41.2, 41.1, 40.7, 40.4, 40.1, 37.0, 36.6, 35.8, 34.7, 32.0, 29.3, 28.7, 24.4, 8.7.

Analogs **2.22-2.31, 2.36-2.40, 3.3, 3.4, 3.6 and 3.12** were obtained as 5 mg portions pre-dissolved in DMSO from ChemBridge corporation and used directly in the [3H]-Asp-uptake assay.

## Abbreviations

CNS: Central nervous system
EAAT: Excitatory amino acid transporter
SAR: Structure-activity-relationship.
